# How Nutritious Are French Beans (*Phaseolus vulgaris* L.) from the Citizen Science Experiment?

**DOI:** 10.3390/plants13020314

**Published:** 2024-01-20

**Authors:** Lovro Sinkovič, Vanja Blažica, Bojan Blažica, Vladimir Meglič, Barbara Pipan

**Affiliations:** 1 Crop Science Department, Agricultural Institute of Slovenia, Hacquetova Ulica 17, SI-1000 Ljubljana, Slovenia; vladimir.meglic@kis.si (V.M.); barbara.pipan@kis.si (B.P.); 2 Lifely s.r.l., Viale Umberto I, 42 07100 Sassari, Italy; vanja@tomappo.com; 3 Proventus d.o.o., Gradišče 20, SI-5270 Ajdovščina, Slovenia; bojan.blazica@ijs.si; 4 Department of Computer Systems, Jozef Stefan Institute, Jamova 39, SI-1000 Ljubljana, Slovenia

**Keywords:** Citizen Science experiment, bean pods, survey of growth habits, food composition database, nutritional quality

## Abstract

French beans are tender, immature, edible pods that are harvested early in the plant’s growth cycle and are usually eaten cooked. The growth habits of French beans were studied for the first time in a Citizen Science experiment, and 19 pod samples were collected for further nutritional analysis. Various macronutrients (e.g., protein, ash, fat, carbohydrates, amino acids) and multi-element profiles were determined. A survey of their growing habits revealed that beans are usually planted once or twice a year in May and June at a length of 5–10 m, with a predominance of dwarf beans cultivation over climbing varieties, and pest resistance and stringless pods are the most important characteristics when deciding on a bean. Homogenised freeze-dried pod samples contained 16.1–23.1% protein, 4.5–8.2% ash, 0.1–1.1% fat, and 62.0–70.6% carbohydrates and had a caloric value of 337–363 kcal/100 g. Of the 17 free amino acids identified, 8 were essential (histidine, threonine, methionine, valine, lysine, isoleucine, leucine, phenylalanine) and 9 were non-essential (cysteine, aspartic acid, serine, glutamic acid, glycine, arginine, alanine, proline, tyrosine); meanwhile, of the 12 elements, 5 were macroelements and 7 were microelements. The predominant free amino acids were aspartic acid, glutamic acid, and serine. In the multiple comparisons (Box and Whisker plot), the parameters caloric value and iron showed the strongest response. A very strong positive significant Pearson correlation (≥0.95) was found for five pairs of variables within the free amino acids. Comparison of the nutrient data obtained in the pods showed near-perfect or high complementarity (85.2–103.4%) with the food composition databases for half of the parameters, suggesting that the home-grown French beans from the Citizen Science experiment are a highly nutritious vegetable.

## 1. Introduction

Common bean (*Phaseolus vulgaris* L.) is one of the most important legumes for direct human consumption and one of the most widely cultivated bean species in the world [1]. It is a multipurpose crop that can be grown and consumed as a dry bean harvested at the full maturity stage, as a shell bean harvested at the physiological maturity stage, and a French bean as a vegetable before seed development [2,3]. French beans (syn. snap beans, green beans, string beans) are common beans grown for their edible, immature, tender pods [4], which have a short growing season and high nutritional value [5]. In general, there are two growth forms of French bean—climbing (pole, indeterminate) and dwarf (bush, determinate)—the latter being more commonly cultivated due to its earlier maturity and the fact that it does not require climbing support [6]. About 150 varieties of French beans, varying in colour, shape, and size, are produced worldwide for domestic consumption and export [7]. There have been two historical milestones in the production and use of French beans, namely the invention of the stringless trait by CN Keeney in 1870 and the breeding of the Blue Lake variety developed for canning and processing in the USA in the early 1900s [3].

French beans develop fresh, fleshy, tender pods with reduced fibre content in the pod wall. The fast-growing green, yellow, and/or purple pods with or without flecks/spots are usually harvested fleshy and tender with small and underdeveloped seeds [8]. The pods of French beans generally contain as little inedible fibre as possible in the pod wall and are consumed as a whole vegetable. Key characteristics of French beans include a reduced fibre content in the pod wall, absence of pod suture strings, and thickened but juicy pod walls [9]. Thermal processing, hereafter referred to as cooking, is a fundamental step in the preparation of French beans for consumption that aims to achieve palatability associated with increased nutrient digestibility, reduction/elimination of antinutrients, and improved sensory characteristics such as aroma, flavour, and texture [10]. From a nutritional point of view, French beans have a high water content and are rich in proteins, vitamins, minerals and dietary fibre. They are also cholesterol-free and have a low sodium and fat content [7]. The consumption of French beans is highly recommended as part of a low-carbohydrate diet, and the low glycaemic index makes them suitable for diabetics [4,11]. The nutritional value of French bean protein depends mainly on its free amino acid content, of which leucine and glutamic acid are present in large quantities [12]. As the most abundant essential amino acids (EAAs), branched-chain amino acids (BCAAs), which include leucine, isoleucine, and valine, are not only the substrates for the synthesis of nitrogen compounds, but also serve as signalling molecules that regulate the metabolism of glucose, lipids, and protein synthesis, gut health, and immunity [13]. The human body requires about twenty essential elements to function properly, as they act as important phytochemicals and play a significant role in maintaining health [14,15]. In addition, seven macroelements are essential: sodium, potassium, magnesium, calcium, chlorine, phosphorus, and sulphur. Furthermore, microelements or trace elements are also defined as essential for plants or animals: manganese, iron, copper, zinc, selenium, cobalt, molybdenum, and iodine [14].

Research projects are increasingly involving the public in data collection, data analysis, and other parts of the scientific process, and such efforts are commonly referred to as Citizen Science experiments. Citizen Science typically involves professional researchers and nonprofessional volunteers working together to help with data collection and other predefined research tasks [16]. Although there is a long history of public engagement in agricultural and food science, these efforts have rarely been referred to as ‘citizen science’. Currently, there are comparatively few new Citizen Science experiments that focus on food or agriculture, and even fewer that target gardeners/farmers or investigate agronomic research questions [17]. A decade ago, the European Citizen Science Association (ECSA) was founded, which emerged from an informal network of researchers and communicators interested in citizen science in Europe (www.ecsa.ngo). The role of Citizen Science in supporting R&I in the European Union has increased in recent years [18]. In Slovenia, an initiative is underway to join the highly dynamic international efforts in the field of Citizen Science by involving citizens in the diversity of research actors, which can improve innovative solutions to problems in the natural and social environment [19].

The intraspecific diversity of French beans is consistently related to growing conditions and differences in the nutrient composition of the pods. Due to the increasing importance of French beans among gardeners and consumers, the nutrient profile of pods of different genotypes collected during the first Citizen Science experiment on this species in Slovenia (Central European growing conditions) was investigated. The French bean pods were analysed for macronutrients (fats, carbohydrates, proteins including amino acid composition) and multi-element profiles. The aim of this study was to obtain data on the growth habits of this nutritious vegetable crop among gardeners and to assess the extent to which the nutrients in the home-grown French beans matched those available in food composition databases (e.g., USDA, EuroFIR). The second objective was to determine whether there were significant differences in nutrients in the pod samples collected that would provide insight into the range of potential nutrient intakes in a diet of produce from home vegetable gardens.

## 2. Results and Discussion

### 2.1. French Bean Growing Habits Survey

**Figure 1:**
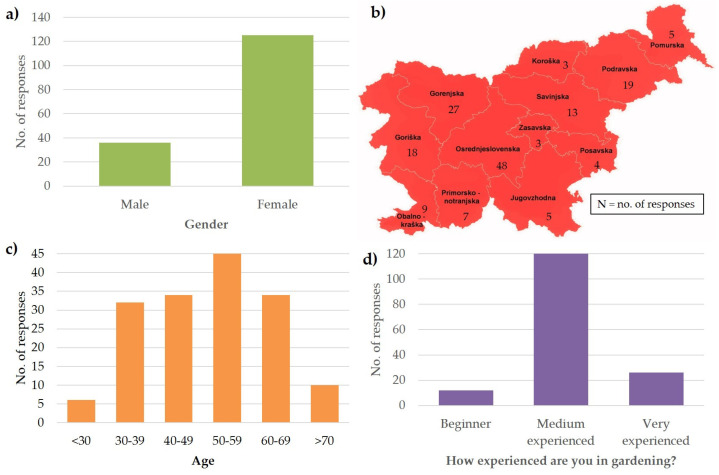
Frequency distribution from the French bean survey for four general aspects: (**a**) gender of respondents, (**b**) distribution of respondents by statistical region, (**c**) age respondents, and (**d**) gardening experience of respondents.

The online survey was sent to Tomappo users, Tomappo newsletter recipients, and Tomappo Facebook followers. Over a period of 100 days, from August to November 2021, a total of 161 participants provided information about their French bean growing habits. The results of this survey showed that 78% of the participants were female and 22% male (Figure 1a). Participants came from all regions of Slovenia, with the largest group (30%) coming from the Osrednjeslovenska region (i.e., central Slovenia) (Figure 1b). A proportion of 28% of participants were between 50 and 59 years old, with other age groups well represented (Figure 1c). The majority of participants (76%) described themselves as medium experienced vegetable gardeners, 16% as very experienced, and 8% as beginners (Figure 1d).

**Figure 2:**
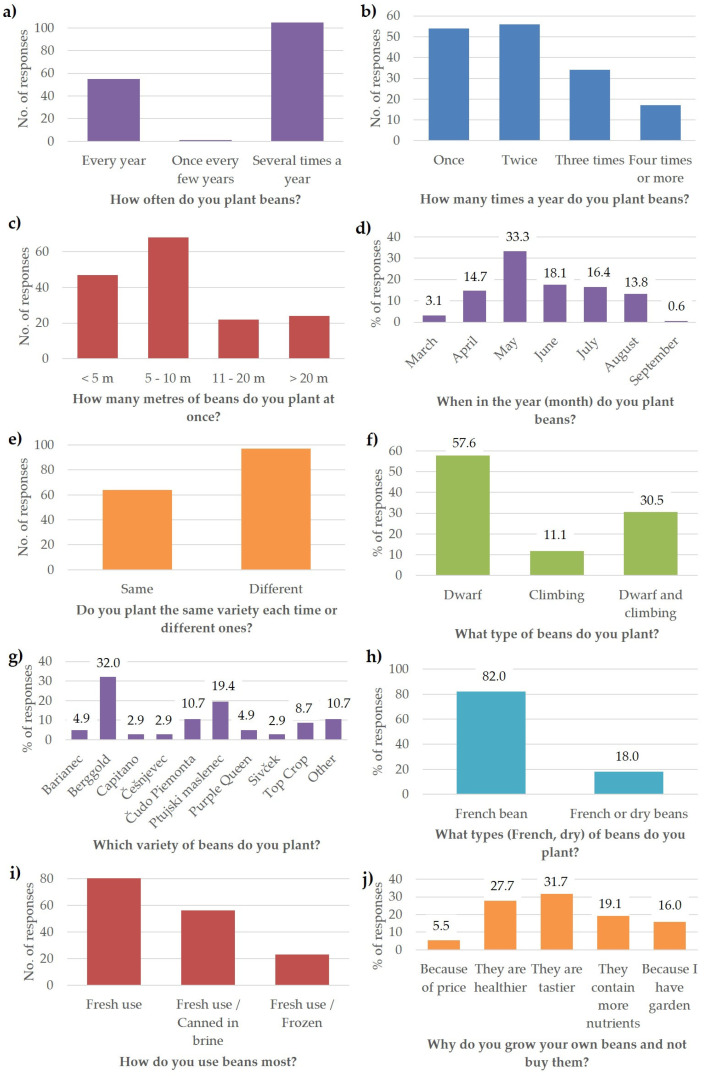
Frequency distribution from the French bean survey for ten aspects of growing habits: (**a**) How often do you plant beans? (**b**) How many times a year do you plant beans? (**c**) How many metres of beans do you plant at once? (**d**) When in the year (month) do you plant beans? (**e**) Do you plant the same variety each time or different ones? (**f**) What types (dwarf, climbing) of beans do you plant? (**g**) Which variety of beans do you plant? (**h**) What types (French, dry) of beans do you plant? (**i**) How do you use beans most? and (**j**) Why do you grow your own beans and not buy them?

The frequency distribution for ten growth habit characteristics based on the French bean survey is summarised in Figure 2. The results show that the majority of respondents (105 or 65%) plant beans several times a year, and 55 of them or 34% plant beans every year (Figure 2a). Most plant beans twice (56 responses), once (54 responses), or three times a year (34 responses) (Figure 2b). Beans are most frequently planted at a length of 5–10 m (68 responses) or <5 m (47 responses) at one time (Figure 2c). Beans are most commonly planted in May (33% of responses), followed by June (18% of responses), July (16% of responses), April (15% of responses), and August (14% of responses); meanwhile, March and September are the months when few people plant beans (3% and 1% of responses, respectively) (Figure 2d). Regarding bean varieties, 60% of respondents plant a different variety each time and 40% plant the same variety (Figure 2e). In terms of growth habit, dwarf beans are the most popular (57.6% of responses), followed by a combination of dwarf and climbing beans (30.5% of responses) and climbing beans (11.9% of responses) (Figure 2f). The most common bean variety is Berggold (32.0% of responses), followed by Ptujski maslenec (19.4% of responses) and Čudo Piemonta (10.7% of responses), also known as Merveille de Piemonte (Figure 2g). Depending on the intended use, most beans are grown as French beans (82.0% of responses) and a smaller proportion for combined use—French or dry beans (18.0% of responses) (Figure 2h). Most beans are consumed fresh in salads, stews, or traditional dishes (82 responses), but the combination of fresh consumption and canning in brine (56 responses) and fresh consumption and freezing (23 responses) is also traditionally common (Figure 2i). Most people believe that home-grown beans are tastier (32% of responses), healthier (28% of responses), and more nutritious (19% of responses), while a smaller proportion only grow beans because they have a garden (16% of responses) or because of the price (5% of responses) (Figure 2f).

**Figure 3:**
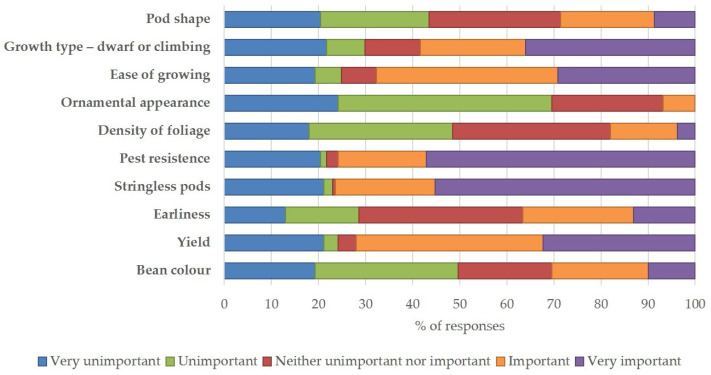
Importance degree of ten categories in the choice of bean variety from the French bean survey.

Figure 3 summarizes the degree of importance of ten categories when deciding on a bean variety for cultivation. The results show that the majority of respondents consider resistance to pests and stringless pods as the most ‘very important’ characteristics (57% and 55% of responses, respectively), followed by growth habit (dwarf or climbing bean) (36% of responses), yield (32% of responses), and ease of cultivation (29% of responses). In addition, the density of foliage and the appearance as an ornamental plant are considered the least ‘very important’ characteristics (≤4% of responses). Among the ‘important’ characteristics, yield and ease of growing ranked first (40% and 39% of responses, respectively), while appearance as an ornamental plant ranked last (7% of responses). Among the characteristics rated as ‘neither unimportant nor important’, earliness, density of foliage, and pod shape (≥28% of responses) ranked high; among the ‘unimportant’ characteristics, ornamental appearance, density of foliage, and bean colour (≥30% of responses) ranked high.

Overall, respondents were very positive about citizen science and considered it a useful approach in agriculture for all survey purposes, especially for data collection. A survey on bean growing habits in Slovenia revealed that the most common month for planting is May, followed by June and July. The majority of respondents plant beans with a length of 5–10 m or up to 5 m once or twice a year. The cultivation of dwarf beans is more widespread compared to climbing beans, with French beans dominating over dry beans. The most widespread dwarf varieties are Berggold and Čudo Piemonta, while the most widespread climbing variety is Ptujski maslenec. The significance of pest resistance and stringless pods are more important characteristics than growth type and yield when selecting bean varieties for cultivation. The data obtained as part of a Citizen Science experiment provides an initial insight into the motivations, priorities, and general cultivation habits of home-grown bean cultivation and is therefore of crucial importance for answering further agricultural research questions on this vegetable species.

### 2.2. Proximate Analysis and Caloric Value of French Beans

The basic chemical composition and caloric value of the French bean pods are shown in Table 1. Significant differences between the samples were found for the parameters water content, protein, ash, and fat. The most pronounced differences between the French bean pods were found in protein (CV = 9.93%), ash (CV = 15.55%), and fat content (CV = 49.35%). The water content of the bean pods ranged from 72.57% in KIS_10 to 93.00% in KIS_12. The lower water content in the pods of sample KIS_10 compared to the other samples tested was most likely due to over-ripeness, as the seeds in the pod were already well-rolled and developed.

The protein content of the French bean pods varied significantly between 16.1 and 23.1 g/100 g DW with an average value of 19.4 g/100 g DW (Table 1). The pod samples KIS_6 and KIS_16 had a high protein content (>22%), while the pods of KIS_1 and KIS_18 had a low protein content (<17%). The ash content varied between 4.72 g/100 g DW for the pod sample KIS_9 and 8.13 g/100 g DW for the pod sample KIS_4. The fat content ranged from 0.1 to 1.2 g/100 g. The pod samples KIS_12 and KIS_15 had a high fat content (>1%), while the group of five samples (KIS_2, KIS_3, KIS_7, KIS_11, and KIS_17) had a low fat content (<0.5%). The range of carbohydrate and energy values was relatively small, with average values of 66.8 g/100 g DW and 351 kcal/100 g DW, respectively. These data are consistent with those reported for common beans (all *Phaseolus vulgaris* L.), which generally contain 16–33% protein, 1–3% fat, and 50–60% carbohydrates [4,10,20,21,22]. Delfini et al. [23] reported a lower range (16.9–20.2 g/100 g) for protein content in seeds from 1512 accessions of Brazilian common bean germplasm compared to our data. The interaction between irrigation and nitrogen application during plant growth has a significant effect on protein content in French bean pods (12–18% DW) [24]. The highest caloric value among the samples was in the pods of KIS_10 and the lowest in the pods of KIS_3, but these differences were not significant.

### 2.3. Amino Acid Profile of French Beans

The free amino acid composition of the French bean pods is shown in Table 2. Seventeen free amino acids were quantified, of which eight were EAAs and the other nine were NEAAs. The EAAs (histidine (*His*), threonine (*Thr*), methionine (*Met*), valine (*Val*), lysine (*Lys*), isoleucine (*lle*), leucine (*Leu*) and phenylalanine (*Phe*)) and the NEAAs (cysteine (*Cys*), aspartic acid (*Asp*), serine (*Ser*), glutamic acid (*Glu*), glycine (*Gly*), arginine (*Arg*), alanine (*Ala*), proline (*Pro*), and tyrosine (*Tyr*)) were detected in the pods of all French bean samples (Table 2). The proportions of EAAs and branched-chain amino acids (BCAAs), which include the essential nutrients leucine (*Leu*), isoleucine (*Ile*), and valine (*Val*), ranged from 23.5 to 36.4% of total protein and from 9.8 to 15.5% of total protein, respectively; meanwhile, the proportions of NEAAs ranged significantly from 43.5 to 56.3% of total protein (Table 2). French bean pod samples KIS_7 and KIS_8 had the highest proportion of NEAAs (>54% of total protein), while the proportion of EAAs was highest in samples KIS-10 and KIS-19 (>31% of total protein). The highest proportion of BCAAs was found in French bean pod samples KIS_1, KIS_6, KIS_8, KIS_10, and KIS_19 (>13% of total protein). The pods of sample KIS_10 had the significantly highest proportion of EAAs *His*, *Lys*, *Ile*, *Leu*, and *Phe*, while *Thr* and *Val* were highest in the pods of sample KIS_1.

The results of free amino acid analysis showed that significant differences in their concentrations in French bean pods could be due genotypic effects between genotypes/varieties [12,25] and growing conditions [26]. Leucine (*Leu*; average 5.11% of total protein) and lysine (*Lys*; average 4.58% of total protein) dominated among the EAAs in French bean pods, while aspartic acid (*Asp*; average 14.43% of total protein), glutamic acid (*Glu*; average 8.92% of total protein), and serine (*Ser*; average 7.40% of total protein) dominated among the NEAAs. In addition, French bean pod samples KIS_10 and KIS_19 had the highest concentrations of *Leu* and *Lys*, KIS_2, KIS_3, KIS_4, and KIS_5 contained the highest concentration of *Asp* (>16% of total protein), while KIS_10 and KIS_19 were richest in *Glu* (>13% of total protein) (Table 2). Fukuji et al. [12] reported higher concentrations of the EAAs *Val*, *Lys*, *Leu*, and *Phe* and the NEAAs *Glu*, *Pro*, and *Try* in French bean genotypes compared with our data, whereas *Asp* and *Ser* were lower. Similarly, El Sheikha et al. [20] reported higher *Lys* and *Arg* content and lower *Asp* and *Ser* content in pods of French bean variety Polista treated with biostimulants during growth. Moreover, in the study by Nemeskéri [26], shelled and dry beans had higher concentrations of the EAA *Phe* and the NEAAs *Glu* and *Tyr*, while *Asp* and *Ser* were also lower. In this context, shelled beans had significantly lower concentrations of *Glu* compared to dry beans, accounting for 3.1–4.1 and 12.9–16.2% of total protein, respectively. Significant differences between the individual free amino acids, EAAs, BCAAs, and NEAAs were found in the French bean pod samples analysed.

**Figure 4:**
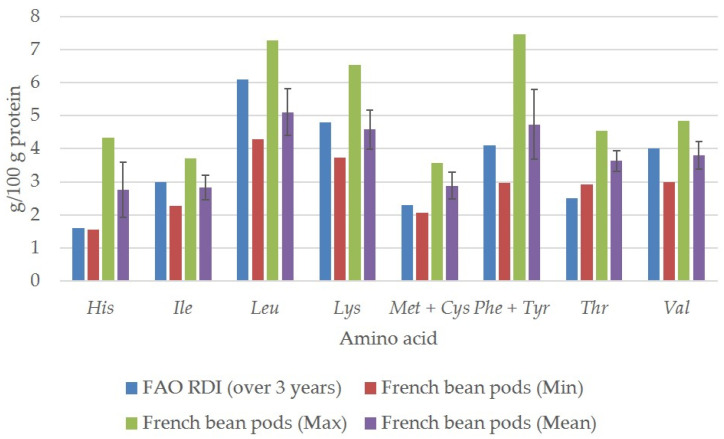
Free amino acid levels in the pods of the French bean samples (*n* = 19) compared to FAO recommended daily amino acid reference values for older children (older than 3 years), adolescents, and adults.

Figure 4 shows the amino acid concentrations in the pods of French bean samples compared to the Food and Agriculture Organisation (FAO) of the United Nations [27] recommendations for the daily intake (RDI) of individual essential amino acids for older children, adolescents, and adults (older than 3 years). Consumption of French beans covers the nutritional requirements for the two essential amino acids histidine and threonine (Figure 4). The average content of histidine (*His*), methionine + cysteine (*Met* + *Cys*), phenylalanine + tyrosine (*Phe* + *Tyr*), and threonine (*Thr*) in the French beans studied can cover the recommended daily intake. However, the average content of isoleucine (*Ile*), leucine (*Leu*), lysine (*Lys*), and valine (*Val*) in the French bean pods studied was below the recommended daily value. Considering that a meal can contain more than 100 g of French beans, these recommendations can easily be met with French beans alone.

### 2.4. Multi-Elemental Profile of French Beans

Twelve elements were quantified in the pods of the French bean samples (Table 3). The elements can be divided into two groups: macroelements (>1 g/kg DW)—Mg, P, S, K, and Ca; microelements (>0.01 mg/kg DW)—Na, Mn, Fe, Co, Cu, Zn, and Mo. The order of elements from most to least abundant (based on mean values) is as follows: K (22.31 g/kg DW), P (4.90 g/kg DW), Ca (4.69 g/kg DW), S (2.45 g/kg DW), Mg (2.26 g/kg DW), Fe (64.59 mg/kg DW), Zn (30.85 mg/kg DW), Na (27.99 mg/kg DW), Mn (17.04 mg/kg DW), Cu (7.12 mg/kg DW), Mo (4.25 mg/kg DW), and Co (0.09 mg/kg DW). The highest coefficient of variation among the macroelements was calculated for K and Ca (>19%) and among the microelements for Co and Mo (>66%). The most abundant element K was significantly higher in the pods of six samples, namely KIS_2-6, KIS_7, and KIS_11 (>25 g/kg DW). The pods of sample KIS_16 contained significantly the highest macroelements Mg, P, and S. Significantly higher Fe contents were found in the pods of samples KIS_9, KIS_15, and KIS_17 and Zn content in KIS_5 (>82 mg/kg DW). El Sheikha et al. [20] studied the French bean variety Polista during two growing seasons using foliar biostimulants and reported lower K, Ca, S, P, Fe, Zn, and Cu contents, while Sheibanirad et al. [28] found lower K content and higher levels of Ca, Mg, Fe, and Zn in the pods of different *Phaseolus vulgaris* varieties in Iran compared to our data. The study on the diversity of elemental content of seeds of 1512 accessions from Brazilian bean germplasm revealed lower mean values for K (17.37 g/kg) and Ca (1.49 g/kg), while the values for Cu (12.55 g/kg) and Fe (80.48 g/kg) were higher compared to our data [23]. In addition, Celmeli et al. [22] reported lower values for Zn (18–38 mg/kg DW) and higher values for Fe (42–134 mg/kg DW) in the seeds of 15 landraces and varieties of common bean. Significant differences were found between the French bean pod samples for all macro- and microelements.

### 2.5. Multivariate Analysis

**Figure 5:**
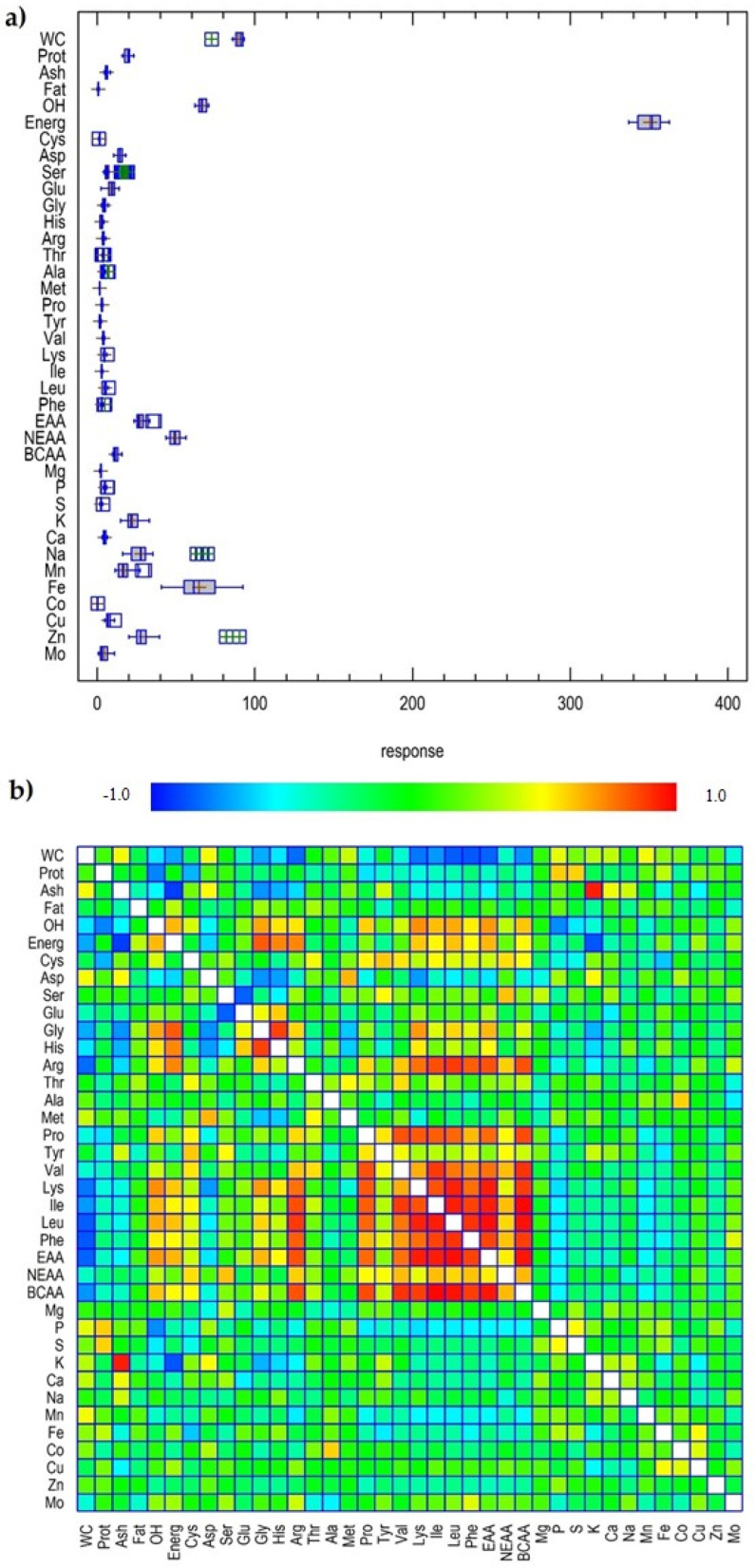
Box and Whisker plot (**a**) and Pearson’s correlation matrix (**b**) for 38 variables studied in French bean pod samples. Legend: WC, water content; Prot, protein; OH, carbohydrates; Energ, caloric value; *Cys*, cysteine; *Asp*, aspartic acid; *Ser*, serine; *Glu*, glutamic acid; *Gly*, glycine; *His*, histidine; *Arg*, arginine; *Thr*, threonine; *Ala*, alanine; *Met*, methinonine; *Pro*, proline; *Tyr*, tyrosine; *Val*, valine; *Lys*, lysine; *Ile*, isoleucine; *Leu*, leucine; *Phe*, phenylalanine; EAA, essential amino acids; NEAA, non-essential amino acids; BSAA, branched-chain amino acids.

Statistical analysis of this data was performed by multiple comparisons to reveal variations among French bean pods in terms of specific nutritional characteristics. This distribution was visualized in the form of a Box and Whisker plot, which can be seen in Figure 5a. Overall, caloric value (Energ) and Fe showed the highest response in all 19 French bean samples analysed. In addition, K, Na, Zn, Mn, and NEAAs showed a moderate response, followed by glutamic acid (*Glu*), Mo, EAAs, carbohydrates (OH), water content (WC), protein (Prot), aspartic acid (*Asp*), Cu, and BCAAs (Figure 5a).

In addition, the matrix of 38 variables for the 19 French bean pod samples was evaluated using Pearson’s rank correlation coefficients to a significance of *p* < 0.05 (Figure 5b). Isoleucine (*Ile*) showed very strong positive correlations with BCAAs, *Leu*, EAAs, and *Phe* (>0.87). Leucine (*Leu*) had very strong positive correlations with EAAs, BCAAs, and *Phe* (>0.91), while EAAs showed a very strong positive correlation with BCAAs (0.95). Lysine (*Lys*) had very strong positive correlation with EAAs, Leu, BCAAs, and Ile (>0.84). Valine (*Val*) showed very strong positive correlations with BCAAs and Ile (>0.89), while *Phe* had very strong positive correlations with BCAAs and EAAs (>0.87). Arginine (*Arg*) showed very strong positive correlations with *Leu*, *Ile*, EAAs, BCAAs, and *Phe* (>0.82), and glycine (*Gly*) had a very strong positive correlation with *His* (0.87). Proline (*Pro*) showed very strong positive correlations with BCAAs, *Val*, *Lys*, *Ile*, *Leu,* and EAAs (≥0.80), whereas serine (*Ser*) had a very strong negative correlation with *His* (−0.81). A very strong correlation was previously reported for *Ile* with *Val*, a strong correlation for *Ile* with *Leu*, and strong correlations for *Phe* with *Ile*, *Leu,* and *Arg* in plants [29]. Similarly, *Asp* showed a strong correlation with *Met* and *Pro* with *Phe* in grass pea [30]. For basic chemical composition, caloric value showed a very strong negative correlation with ash (−0.88) and a very strong positive correlation with *Gly* (0.82). Water content (WC) showed a very strong positive correlations with *Leu*, *Phe*, and EAAs (≥0.80). Finally, K showed a very strong positive correlation with ash (0.94) and a very strong negative correlation with caloric value (−0.83), whereas P showed a strong negative correlation with carbohydrates (−0.72). A strong correlation between ash and K was also found for seeds of grain legumes [31].

### 2.6. Complementarity of Results with Food Composition Databases

To assess the extent to which the nutrients determined in home-grown French beans as part of the Citizen Science experiment match the nutrients available in food composition databases, a comparison was made of the data available in the US Department of Agriculture (USDA) [32] and the European Food Information Resource (EuroFIR) [33]. The nutrient comparison of 19 French bean pod samples studied with data from the USDA and EuroFIR databases is summarised in Table 4. In these food composition databases, data were mainly available for ‘proximates’, i.e., water, protein, ash, fat, carbohydrates, and energy, and ‘minerals’, i.e., Mg, P, Ca, K, Na, Mn, Fe, Cu, Zn, and Mo; meanwhile, data on amino acid composition were partially available in the EuroFIR database only in the case of Denmark and Slovenia, so they cannot be objectively compared with the data obtained in this study. Complementarity was calculated as the percentage agreement between the study results and the data from food composition databases. The comparison showed that, for 6 out of 16 parameters, i.e., water, carbohydrates, Mg, Ca, K, and Cu, the complementarity of the study results with the food composition databases was almost perfect, ranging from 96.7 to 103.4% (Table 4). For protein and Zn, the complementarity of the study results with the food composition databases was very high at 90.0 and 85.2%, respectively. For ash (83.0%), Mn (69.0%), and Fe (69.8%), the complementarity between the study results and the food composition databases was slightly underestimated, while it was slightly overestimated for energy (120.5%) and P (124.2%). The parameters fat and Mo achieved a lower complementarity between the study results and the food composition databases (25.9 and 21.3%, respectively), while Na showed the lowest complementarity (8.6%).

## 3. Materials and Methods

### 3.1. Tomappo Survey and Plant Material

To collect samples of home-grown French beans, the community of amateur gardeners around the gardening app Tomappo (version 4.6.1) was approached and the data in the app were examined. Tomappo is a freely available Android and web app (https://tomappo.com) that includes garden information on vegetables and growing methods, a sowing calendar, a weather forecast, a garden planner for designing garden beds throughout the year (taking into account crop rotation and co-planting), and a seed exchange platform. The latter was used to launch a Citizen Science experiment for the first time in Slovenia in 2021 with an online survey among Tomappo users about their French bean growing habits. The survey, which received 161 responses, covered the following aspects: gender (male/female); statistical region (Gorenjska/Goriška/Jugovzhodna/Koroška/Obalno-kraška/Osrednjeslovenska/Podravska/Pomurska/Posavska/Primorsko-notranjska/Savinjska/Zasavska); age (<30/30–39/40–49/50–59/60–69/>70); “How experienced are you in gardening?” (beginner/medium experienced/very experienced); “How often do you plant beans?” (several times a year/every year/once every few years); “How many times a year do you plant beans?”; “How many metres of beans do you plant at once?”; “When in the year (month) do you plant beans?” (March–September); “Do you plant the same variety each time or different ones?” (same/different); “What types (dwarf/climbing; French/dry) or varieties of beans do you plant?”; “How do you use beans most?” (fresh use/frozen/canned in brine); “Why do you grow your own beans and not buy them?” (because of price/homegrown beans are healthier/homegrown beans are tastier/homegrown beans contain more nutrients/because I have garden); “Rate how important each of these categories is to you when choosing a variety” (very unimportant, unimportant, neither unimportant nor important, important, very important)—bean colour, yield, earliness, stringless pods, pest resistance, density of foliage, ornamental appearance, ease of growing, growth type (dwarf or climbing), and pod shape.

**Figure 6:**
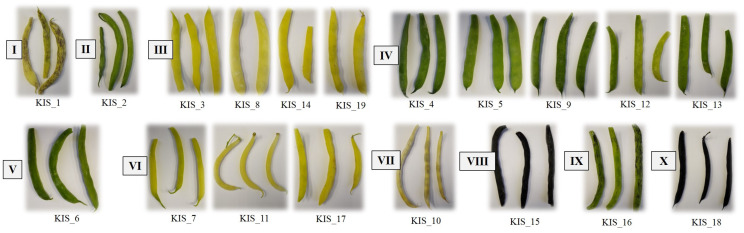
Classification of the French beans studied into ten groups based on pod colour and shape of pod cross-section. (**I**) Yellow with violet flecks/narrow elliptic; (**II**) green/circular; (**III**) yellow/narrow elliptic; (**IV**) green/narrow elliptic; (**V**) green/elliptic–ovate; (**VI**) yellow/elliptic–ovate; (**VII**) yellow/circular; (**VIII**) violet/elliptic–ovate; (**IX**) green with violet flecks/narrow elliptic; (**X**) violet/narrow elliptic.

In addition, the Tomappo users were asked to provide pods of each variety from their gardens for a comprehensive characterisation of the nutritional properties. Finally, the thirteen users provided 19 samples of French beans (*Phaseolus vulgaris* L.) from different locations in Slovenia (Table 5). The respondents who were willing to donate French bean samples informed us, and sampling was carried out at the stage of technological pod maturity under our supervision according to the standard procedure for bean pods. The 10–15 fresh bean pods from several plants—collected from each donator to capture as much variability as possible—were brought to the laboratory immediately after harvest in a portable cool box, photographed, and frozen at −20 °C until freeze-drying was conducted. These pod samples were classified into ten groups based on pod colour and pod cross-section shape using descriptors for *Phaseolus vulgaris* based on the pods defined by the Community Plant Variety Office [34] (Figure 6). Five samples had green pods with a narrow elliptical cross-section, four samples had yellow pods with a narrow elliptical cross-section, and three samples had yellow pods with an elliptical–ovate cross-section. The other seven samples differed in colour and shape: yellow with violet flecks/narrow elliptic, green/circular, green/elliptic–ovate, yellow/circular, violet/elliptic–ovate, green with violet flecks/narrow elliptic, and violet/narrow elliptic (Figure 6). The bulk sample from eight most representative pods were freeze-dried, pulverised, and homogenised with a laboratory ball mill (Retsch MM 400, GmbH, Haan, Germany) at a high frequency of 30 Hz for 2–4 min before further nutritional analyses were performed.

### 3.2. Proximate Analysis and Caloric Value

Chemical composition was determined for all homogenised samples, i.e., determination of the dry matter; determination of the protein, ash, and fat content; calculation of the water content, total carbohydrates, and caloric value. Dry matter content was determined through drying the freeze-dried samples at 103 °C for 48 h according to the standard method [35] in the performance oven (SP-105 C, Kambič, Semič, Slovenia). Crude protein was analysed according to the Kjeldahl method [36] with a factor of 6.25 on a Kjeltec 8400 (Foss, Hoganas, Sweden). Ash content was determined based on the weight difference before and after combustion at 550 °C for 4 h [37] in an oven (Aurodent G-9-1206 B, Celje, Slovenia). Crude fat was analysed by extraction with petroleum ether according to the standard method [38] using an extraction system (Buchi B-811, Uster, Switzerland). The chemical composition was determined in triplicate and the results are expressed as a percentage (%) on a dry matter basis. The water content (%) was calculated from the dry matter (%) and the difference between the masses before and after freeze-drying. Total carbohydrates = 100 − (% protein + % fat + % ash + % moisture) [39]. The caloric value (kcal/g) of the pods was calculated using the calorie conversion factors for proteins (4 kcal/g), fats (9 kcal/g), and carbohydrates (4 kcal/g) [40].

### 3.3. Amino Acid Analysis Using HPLC

The content of free amino acids was determined according to the modified method ISO 13903 [41] for plant material (internal laboratory protocol) after acid hydrolysis. Prior to the hydrolysis step, cysteine and methionine were oxidised to cysteic acid and methionine sulphone. The oxidised samples were hydrolysed with 6 M hydrochloric acid containing phenol for 24 h. Amino acids were derivatised using AccQ Tag Reagents (N-aminoquinolyl succinate, Waters, Milford, MA, USA). High-performance liquid chromatography with fluorescence detector (HPLC-FLD, Agilent 1200, Agilent Technologies, Santa Clara, CA, USA) was used for the analysis. The amino acids were separated on a NovaPak C-18 column (150 × 3.9 mm, 5 µm; Waters, Milford, MA, USA) using a triple gradient of phosphate buffer (pH 5.05), acetonitrile, and water at 28 °C, and a flow rate of 1 mL/min. The injection volume was 10 µL. The fluorescence detector operated at λ(ex) = 250 nm and λ(ex) = 395 nm. A mixture of amino acid standard (CAS No. AAS18, Supelco, Merck KGaA, Darmstadt, Germany) with 17 amino acids, cysteic acid monohydrate (Merck-Aldrich), methionine sulfone (Supelco), and alpha-butyric acid (Supelco) was used as an internal standard to calibrate the HPLC. Daily calibration was performed with an internal standard at one concentration. Linearity, precision, accuracy, and LOQ were determined during validation. The amino acid composition was determined in triplicate and the results are reported as a percentage of total proteins on a dry matter basis (% of total proteins).

### 3.4. Multi-Elemental Analysis Using ICP-MS

Mg, P, S, K, Ca, Na, Mn, Fe, Co, Cu, Zn, and Mo were analysed by inductively coupled plasma mass spectrometry (ICP-MS). Homogenized samples (250 mg) were mixed with 6 mL nitric acid (65%, *v/v*; Suprapur, Merck KGaA, Darmstadt, Germany) and 2 mL hydrogen peroxide (30%, *v/v*; Suprapur, Merck) and digested using the Milestone Ethos 1600 microwave digestion system. The digested solutions were cooled to room temperature and diluted to 50 mL with double-deionized water (resistivity of 18.2 MΩ: Millipore, Bedford, MA, USA). Before analysis, the digested samples were diluted by a factor of 20 and consisted of 1% (*v/v*) nitric acid. The content of elements in the samples was determined using an Agilent ICP-MS 7900 (Tokyo, Japan) with a 4^th^-generation collision/reaction cell, Octopole Reaction System (ORS^4^). Helium (He) was used as the reaction gas at a flow rate of 5 mL/min in He mode and 10 mL/min in HEHe mode. The isotopes monitored were ^23^Na, ^24^Mg, ^31^P, ^34^S, ^39^K, ^43^Ca, ^55^Mn, ^56^Fe, ^59^Co, ^63^Cu, ^66^Zn, and ^95^Mo. The majority of the elements were measured in the He mode, while P and S were measured in the HEHe mode. The calibration curve was prepared using IV-STOCK-50 standard solution (Inorganic Ventures, Christiansburg, VA, USA) and single standard solutions of P and S (Inorganic Ventures, Christiansburg, VA, USA) were added separately to the mixture. Certified reference materials (NIST SRM 1573a tomato leaves and SRM 1547 peach leaves, Gaithersburg, MD, USA) were used to verify the accuracy of the results. All sample results are reported on a dry matter basis.

### 3.5. Data Analysis

Statistical calculations including ANOVA (Fisher’s least significant difference; *p* < 0.05) and multivariate analysis were performed using the program Statgraphics Centurion v18.1.16 (StatPoint Technologies, Inc., Warrenton, VA, USA). Multiple sample comparisons were calculated to show variation within the French beans studied by specific nutritional characteristics. This distribution was visualized as a Box and Whisker plot. Pairwise associations between individual nutritional traits were assessed using Pearson correlation analysis (1.0 = |r|, perfect correlation; 0.8 < |r| < 1.0, very strong correlation; 0.6 < |r| < 0.8, strong correlation; 0.4 < |r| < 0.6, moderately strong correlation).

## 4. Conclusions

This study is the first application of the Citizen Science experiment to evaluate the growth habits of French beans (*Phaseolus vulgaris* L.) and analyse their nutritional quality. An online survey on growth habits revealed that the majority of respondents plant the beans once or twice a year at a length of 5–10 m, usually in May and June. Dwarf bean cultivation outweighs climbing bean cultivation, with Berggold, Ptujski maslenec, and Čudo Piemonta varieties dominating. Pest resistance and stringless pods were the most important characteristics when deciding on a bean variety for cultivation. Significant differences were found between French bean pods for most macronutrients (water, protein, ash, fat, amino acids) and macro- and microelements studied. The determined nutrient profiles were compared with food composition databases (FCDs), and complementarity was almost perfect for 6 out of 16 parameters. All these results confirm the high nutritional value of French beans, provide useful information for breeding programmes, and confirm that the Citizen Science approach (as a part of an open science approach) can be a good support in answering targeted agricultural science research questions.

## Figures and Tables

**Table 1:** Basic chemical composition and caloric value of pods of French beans studied.

Sample	Water Content	Protein	Ash	Fat	Carbohydrates	Caloric Value
	%	g/100 g DW	g/100 g DW	g/100 g DW	g/100 g DW	kcal/100 g DW
KIS_1	91.34 ^a^	16.59 ^e^	6.38 ^c–e^	0.63 ^fg^	68.05	344.23
KIS_2	91.65 ^a^	20.33 ^a–d^	6.53 ^b–d^	0.10 ^j^	65.11	342.67
KIS_3	92.10 ^a^	20.36 ^a–d^	7.42 ^ab^	0.49 ^h^	62.88	337.32
KIS_4	92.38 ^a^	20.69 ^a–c^	8.13 ^a^	0.91 ^bc^	62.11	339.41
KIS_5	91.53 ^a^	19.51 ^b–e^	6.49 ^b–e^	0.71 ^ef^	64.58	342.75
KIS_6	86.13 ^ab^	22.79 ^a^	5.13 ^fg^	0.73 ^ef^	65.73	360.64
KIS_7	92.28 ^a^	18.98 ^b–e^	7.14 ^a–c^	0.10 ^j^	66.06	341.06
KIS_8	88.42 ^a^	17.31 ^de^	5.53 ^e–g^	0.98 ^b^	69.76	357.07
KIS_9	86.84 ^ab^	20.07 ^a–d^	4.72 ^g^	0.70 ^ef^	68.71	361.37
KIS_10	72.57 ^b^	18.65 ^c–e^	5.14 ^fg^	0.93 ^bc^	69.78	362.11
KIS_11	90.23 ^a^	17.64 ^c–e^	7.40 ^ab^	0.23 ^i^	69.00	348.66
KIS_12	93.00 ^a^	20.48 ^a–d^	5.36 ^fg^	1.11 ^a^	65.06	352.21
KIS_13	91.08 ^a^	17.41 ^de^	5.70 ^d–g^	0.62 ^fg^	68.11	347.61
KIS_14	88.64 ^a^	18.57 ^c–e^	5.09 ^fg^	0.75 ^de^	69.41	358.71
KIS_15	92.57 ^a^	20.23 ^a–d^	5.90 ^d–f^	1.13 ^a^	64.55	349.31
KIS_16	92.53 ^a^	22.79 ^a^	5.55 ^d–g^	0.85 ^cd^	63.32	352.08
KIS_17	87.92 ^a^	22.09 ^ab^	5.26 ^fg^	0.15 ^ij^	66.09	354.10
KIS_18	88.78 ^a^	16.33 ^e^	5.69 ^d–g^	0.89 ^bc^	70.51	355.41
KIS_19	86.26 ^ab^	17.42 ^de^	5.32 ^fg^	0.53 ^gh^	70.45	356.20
Range	72.56–93.00	16.09–23.07	4.52–8.21	0.09–1.20	62.01–70.57	337.05–362.72
Mean ± SD	89.28 ± 4.52	19.38 ± 1.92	5.99 ± 0.93	0.66 ± 0.33	66.80 ± 2.63	350.68 ± 7.62
CV (%)	5.07	9.93	15.55	49.35	3.93	2.17

Data are means (*n* = 3). Mean values with different letters (a–j) in a column are significantly different (*p* < 0.05). DW—dry weight.

**Table 2:** Composition of free amino acids in the pods of the French beans studied.

**Sample**	**Essential Amino Acids (% of Total Protein)**								**ΣEAA (% of Total Protein)**	**ΣBCAA (% of Total Protein)**
	***His***	***Thr***	***Met***	***Val***	***Lys***	***Ile***	***Leu***	***Phe***		
KIS_1	1.87 ^f–h^	4.49 ^a^	1.57 ^a–d^	4.69 ^a^	5.10 ^bc^	3.27 ^ab^	5.64 ^b–e^	2.99 ^d–f^	29.60 ^b–d^	13.60 ^ab^
KIS_2	1.87 ^f–h^	3.53 ^bc^	1.75 ^a^	3.96 ^b–e^	3.84 ^gh^	2.75 ^d–f^	4.64 ^f–h^	3.15 ^cd^	25.51 ^c–e^	11.36 ^d–g^
KIS_3	1.75 ^gh^	3.93 ^ab^	1.68 ^ab^	3.93 ^b–e^	4.10 ^f–h^	2.67 ^e–g^	4.63 ^f–h^	2.98 ^d–f^	25.66 ^c–e^	11.23 ^d–g^
KIS_4	1.71 ^gh^	3.84 ^b^	1.56 ^a–d^	3.40 ^e–g^	3.77 ^h^	2.46 ^fg^	4.35 ^h^	2.57 ^e–h^	23.65 ^e^	10.21 ^fg^
KIS_5	1.67 ^h^	3.74 ^b^	1.52 ^a–d^	3.48 ^d–g^	3.90 ^gh^	2.51 ^e–g^	4.51 ^gh^	2.39 ^hi^	23.71 ^e^	10.50 ^fg^
KIS_6	2.25 ^f^	3.53 ^bc^	1.62 ^a–c^	4.11 ^a–d^	4.85 ^b–f^	3.24 ^a–c^	5.81 ^b–d^	3.59 ^bc^	28.99 ^b–d^	13.16 ^a–d^
KIS_7	1.57 ^h^	3.49 ^bc^	1.42 ^c–f^	3.84 ^b–f^	4.56 ^c–g^	2.69 ^e–g^	4.93 ^e–h^	2.91 ^d–g^	25.41 ^c–e^	11.46 ^d–g^
KIS_8	2.16 ^fg^	3.75 ^b^	1.62 ^a–c^	4.22 ^a–c^	4.93 ^b–d^	3.28 ^ab^	5.95 ^bc^	3.82 ^b^	29.74 ^bc^	13.45 ^a–c^
KIS_9	3.42 ^b–e^	3.47 ^bc^	1.48 ^b–e^	3.18 ^g^	4.27 ^d–h^	2.57 ^e–g^	4.81 ^e–h^	2.83 ^d–h^	26.03 ^c–e^	10.56 ^fg^
KIS_10	4.17 ^a^	3.76 ^b^	1.26 ^ef^	4.35 ^ab^	6.10 ^a^	3.61 ^a^	6.99 ^a^	4.66 ^a^	34.90 ^a^	14.95 ^a^
KIS_11	3.46 ^b–d^	3.05 ^c^	1.36 ^d–f^	3.49 ^d–g^	4.88 ^b–e^	2.64 ^e–g^	4.98 ^d–h^	2.90 ^d–g^	26.75 ^b–e^	11.11 ^e–g^
KIS_12	3.26 ^c–e^	3.55 ^bc^	1.44 ^b–f^	3.62 ^c–g^	4.38 ^c–h^	2.62 ^e–g^	4.66 ^f–h^	2.67 ^d–h^	26.20 ^c–e^	10.90 ^fg^
KIS_13	2.98 ^e^	3.63 ^bc^	1.38 ^c–f^	3.47 ^e–g^	4.18 ^d–h^	2.56 ^e–g^	4.40 ^h^	2.53 ^f–g^	25.13 ^de^	10.44 ^fg^
KIS_14	3.62 ^bc^	3.96 ^ab^	1.69 ^ab^	4.22 ^a–c^	4.80 ^b–f^	3.18 ^a–d^	5.47 ^b–f^	2.88 ^d–h^	29.82 ^bc^	12.87 ^b–e^
KIS_15	3.15 ^c–e^	3.48 ^bc^	1.46 ^b–f^	3.29 ^fg^	4.13 ^e–h^	2.28 ^g^	4.38 ^h^	1.96 ^i^	24.13 ^e^	9.95 ^g^
KIS_16	3.14 ^de^	3.57 ^bc^	1.45 ^b–f^	3.41 ^e–g^	4.25 ^d–h^	2.45 ^fg^	4.53 ^gh^	2.43 ^g–i^	25.22 ^de^	10.39 ^fg^
KIS_17	3.41 ^b–e^	3.12 ^c^	1.23 ^f^	3.70 ^c–g^	4.67 ^c–f^	2.80 ^c–f^	5.11 ^c–h^	3.05 ^de^	27.09 ^b–e^	11.60 ^c–g^
KIS_18	3.18 ^c–e^	3.59 ^bc^	1.40 ^c–f^	3.74 ^b–g^	4.90 ^b–d^	2.94 ^b–e^	5.26 ^b–g^	3.09 ^cd^	28.10 ^b–e^	11.94 ^b–f^
KIS_19	3.80 ^ab^	3.56 ^bc^	1.27 ^ef^	4.17 ^a–c^	5.47 ^ab^	3.21 ^a–d^	6.10 ^b^	3.59 ^bc^	31.17 ^ab^	13.48 ^a–c^
Range	1.55–4.34	2.93–4.54	1.20–1.80	3.00–4.85	3.74–6.53	2.28–3.70	4.29–7.28	1.93–4.80	23.46–36.38	9.80–15.46
Mean ± SD	2.76 ± 0.83	3.63 ± 0.32	1.48 ± 0.15	3.80 ± 0.41	4.58 ± 0.59	2.83 ± 0.37	5.11 ± 0.71	3.00 ± 0.59	27.20 ± 2.89	11.75 ± 1.42
CV (%)	30.05	8.83	10.11	10.77	12.92	12.91	13.88	19.70	10.64	12.06
	**Non-Essential Amino Acids (% of Total Protein)**									**ΣNEAA (% of Total Protein)**
	***Cys***	***Asp***	***Ser***	***Glu***	***Gly***	***Arg***	***Ala***	***Pro***	***Tyr***	
KIS_1	1.63 ^ab^	13.28 ^f–j^	6.52 ^de^	9.97 ^d–f^	3.43 ^hi^	3.79 ^e–i^	4.46 ^b–d^	3.68 ^a^	2.32 ^ab^	49.09 ^a–d^
KIS_2	1.3 ^de^	16.87 ^a–c^	7.12 ^d^	10.29 ^c–f^	2.77 ^i^	3.15 ^jk^	4.34 ^c–e^	2.82 ^d–g^	1.62 ^de^	50.33 ^a–d^
KIS_3	1.56 ^a–d^	17.24 ^ab^	6.38 ^d–f^	11.78 ^a–c^	2.74 ^i^	3.30 ^h–k^	4.17 ^c–e^	2.88 ^c–g^	2.39 ^a^	52.44 ^a–c^
KIS_4	1.47 ^b–e^	17.71 ^a^	6.09 ^d–h^	5.99 ^i^	3.05 ^i^	3.09 ^jk^	4.43 ^b–d^	2.66 ^e–g^	1.60 ^de^	46.10 ^b–d^
KIS_5	1.46 ^b–e^	16.43 ^a–d^	6.10 ^d–g^	7.20 ^hi^	3.09 ^i^	3.28 ^h–k^	4.34 ^c–e^	2.64 ^fg^	1.52 ^d–f^	46.06 ^b–d^
KIS_6	1.46 ^b–e^	14.21 ^d–h^	16.97 ^b^	3.35 ^j^	4.14 ^f–h^	4.81 ^ab^	3.62 ^e^	3.14 ^b–e^	2.08 ^bc^	53.80 ^ab^
KIS_7	1.63 ^ab^	14.96 ^b–g^	19.06 ^a^	2.48 ^j^	3.88 ^gh^	3.55 ^f–j^	4.40 ^cd^	3.11 ^b–f^	2.44 ^a^	55.51 ^a^
KIS_8	1.60 ^a–c^	15.40 ^a–f^	15.25 ^c^	3.59 ^j^	4.25 ^e–g^	4.61 ^a–c^	3.92 ^c–e^	3.33 ^a–c^	2.21 ^ab^	54.14 ^ab^
KIS_9	1.27 ^e^	15.11 ^b–g^	4.68 ^h^	10.68 ^b–e^	5.47 ^b–d^	3.94 ^d–g^	3.79 ^de^	2.52 ^g^	1.18 ^gh^	48.65 ^a–d^
KIS_10	1.45 ^b–e^	11.12 ^j^	5.72 ^d–h^	13.25 ^a^	6.48 ^a^	5.14 ^a^	4.31 ^c–e^	3.43 ^ab^	1.79 ^cd^	52.68 ^a–c^
KIS_11	1.39 ^c–e^	12.87 ^g–j^	4.78 ^gh^	8.40 ^gh^	4.27 ^e–g^	3.39 ^g–k^	3.99 ^c–e^	3.17 ^b–d^	2.24 ^ab^	44.50 ^cd^
KIS_12	1.38 ^c–e^	12.25 ^h–j^	5.69 ^e–h^	9.16 ^e–g^	4.92 ^de^	3.24 ^h–k^	4.56 ^bc^	2.74 ^d–g^	1.26 ^f–h^	45.19 ^cd^
KIS_13	1.33 ^de^	15.94 ^a–e^	5.64 ^e–h^	8.07 ^gh^	3.88 ^gh^	2.92 ^k^	6.35 ^a^	2.81 ^d–g^	1.65 ^de^	48.58 ^a–d^
KIS_14	1.51 ^a–d^	14.78 ^c–g^	4.81 ^gh^	10.33 ^c–f^	5.14 ^cd^	4.33 ^b–e^	6.89 ^a^	3.13 ^bf^	1.19 ^gh^	52.12 ^a–c^
KIS_15	0.93 ^f^	13.21 ^f–j^	5.87 ^d–h^	10.22 ^c–f^	4.75 ^d–f^	3.10 ^jk^	5.16 ^b^	2.78 ^d–g^	1.06 ^h^	47.07 ^b–d^
KIS_16	0.86 ^f^	13.19 ^f–j^	5.14 ^e–h^	8.80 ^fg^	4.12 ^f–h^	3.20 ^i–k^	4.54 ^b–d^	2.83 ^d–g^	1.23 ^f–h^	43.91 ^d^
KIS_17	0.90 ^f^	13.90 ^e–i^	4.98 ^f–h^	10.78 ^b–d^	5.15 ^cd^	4.42 ^b–d^	3.99 ^c–e^	2.87 ^c–g^	1.42 ^e–g^	48.42 ^a–d^
KIS_18	1.72 ^a^	13.89 ^e–i^	4.80 ^gh^	11.96 ^ab^	5.85 ^a–c^	3.83 ^d–h^	4.36 ^c–e^	3.17 ^b–d^	1.79 ^cd^	51.36 ^a–d^
KIS_19	1.67 ^ab^	11.80 ^ij^	4.95 ^gh^	13.18 ^a^	5.96 ^ab^	4.04 ^c–f^	4.06 ^c–e^	3.36 ^a–c^	2.05 ^bc^	51.08 ^a–d^
Range	0.86–1.78	10.33–18.06	4.39–19.41	2.43–13.91	2.71–6.93	2.91–5.33	3.58–7.28	2.33–3.68	1.04–2.67	43.50–56.27
Mean ± SD	1.40 ± 0.25	14.43 ± 1.86	7.40 ± 4.30	8.92 ± 3.10	4.39 ± 1.09	3.74 ± 0.65	4.51 ± 0.81	3.00 ± 0.31	1.74 ± 0.47	49.53 ± 3.56
CV (%)	17.90	12.87	58.14	34.79	24.79	17.35	17.88	10.40	27.23	7.18

Data are means (*n* = 3). Mean values with different letters (a–k) in a column are significantly different (*p* < 0.05).

**Table 3:** Multi-elemental composition of pods of French beans studied.

Sample	Macroelement (g/kg DW)					Microelement (mg/kg DW)						
	Mg	P	S	K	Ca	Na	Mn	Fe	Co	Cu	Zn	Mo
KIS_1	2.32 ^a–e^	4.65 ^d–g^	2.67 ^b–e^	24.78 ^cd^	4.36 ^f–h^	33.63 ^b^	13.17 ^ij^	76.10 ^b–d^	0.16 ^b^	8.09 ^cd^	26.54 ^d–h^	2.16 ^ij^
KIS_2	2.08 ^d–f^	4.85 ^c–g^	2.74 ^bc^	25.82 ^bc^	3.48 ^ij^	23.34 ^de^	22.34 ^c^	58.36 ^fg^	0.09 ^d–f^	8.12 ^cd^	27.63 ^d–g^	6.49 ^de^
KIS_3	1.92 ^fg^	5.60 ^a–c^	2.31 ^d–h^	28.94 ^ab^	4.68 ^e–g^	26.64 ^cd^	16.32 ^f–h^	58.08 ^fg^	0.13 ^c^	6.47 ^ef^	23.85 ^e–h^	3.14 ^g^
KIS_4	1.96 ^e–g^	5.75 ^ab^	2.49 ^c–f^	31.45 ^a^	5.59 ^a–c^	30.46 ^bc^	15.24 ^g–i^	60.39 ^ef^	0.09 ^ef^	4.99 ^h^	22.21 ^gh^	2.26 ^hi^
KIS_5	2.09 ^d–f^	5.33 ^b–e^	2.24 ^f–i^	25.48 ^b–d^	4.73 ^d–g^	17.72 ^f^	18.32 ^d–f^	47.92 ^g–i^	0.10 ^de^	6.25 ^fg^	85.97 ^a^	2.63 ^g–i^
KIS_6	2.60 ^a^	4.66 ^d–g^	2.69 ^b–d^	16.59 ^gh^	5.13 ^b–f^	17.11 ^f^	13.84 ^h–j^	69.29 ^de^	0.06 ^g–i^	10.73 ^a^	29.16 ^c–e^	7.38 ^c^
KIS_7	2.50 ^a–c^	4.32 ^fg^	2.99 ^ab^	25.66 ^b–d^	5.71 ^ab^	27.52 ^cd^	17.10 ^e–g^	45.34 ^hi^	0.05 ^h–j^	6.03 ^f–h^	28.56 ^de^	6.12 ^ef^
KIS_8	2.52 ^ab^	4.44 ^fg^	2.00 ^hi^	20.41 ^ef^	5.11 ^b–f^	20.72 ^ef^	19.31 ^de^	54.05 ^f–h^	0.07 ^fg^	6.62 ^ef^	29.42 ^c–e^	5.79 ^ef^
KIS_9	2.16 ^b–f^	4.87 ^c–g^	2.28 ^e–i^	15.59 ^h^	3.33 ^j^	21.27 ^ef^	13.36 ^ij^	87.89 ^a^	0.05 ^h–j^	9.36 ^b^	31.52 ^cd^	10.40 ^a^
KIS_10	2.11 ^d–f^	4.21 ^g^	2.34 ^c–h^	20.75 ^ef^	3.76 ^h–j^	30.73 ^bc^	12.04 ^j^	42.81 ^i^	0.02 ^k^	6.37 ^e–g^	24.23 ^e–h^	7.09 ^cd^
KIS_11	2.58 ^a^	4.42 ^fg^	1.90 ^i^	27.61 ^bc^	6.03 ^a^	66.57 ^a^	12.27 ^j^	54.37 ^f–h^	0.08 ^f^	5.18 ^gh^	25.93 ^e–h^	9.18 ^b^
KIS_12	2.39 ^a–d^	5.37 ^b–d^	2.45 ^c–g^	17.43 ^f–h^	5.24 ^b–e^	27.44 ^cd^	28.70 ^a^	73.16 ^cd^	0.03 ^jk^	5.59 ^f–h^	26.75 ^d–h^	2.33 ^g–i^
KIS_13	2.25 ^a–f^	4.53 ^e–g^	2.07 ^g–i^	20.92 ^ef^	5.50 ^a–d^	26.74 ^cd^	25.64 ^b^	69.62 ^de^	0.35 ^a^	9.97 ^ab^	26.31 ^d–h^	1.90 ^i–k^
KIS_14	1.60 ^g^	5.03 ^b–f^	2.23 ^f–i^	19.93 ^e–g^	3.82 ^h–j^	30.34 ^bc^	12.78 ^ij^	71.22 ^de^	0.14 ^c^	6.54 ^ef^	22.59 ^f–h^	1.82 ^i–l^
KIS_15	2.57 ^a^	4.80 ^c–g^	2.70 ^b–d^	21.91 ^de^	4.86 ^c–g^	29.51 ^bc^	21.21 ^cd^	84.79 ^ab^	0.06 ^gh^	6.40 ^ef^	34.54 ^bc^	1.00 ^l^
KIS_16	2.59 ^a^	6.40 ^a^	3.39 ^a^	20.57 ^ef^	4.25 ^g–i^	31.19 ^bc^	18.45 ^d–f^	75.88 ^b–d^	0.06 ^g–i^	6.74 ^ef^	37.53 ^b^	1.26 ^kl^
KIS_17	2.12 ^c–f^	4.96 ^b–g^	2.68 ^b–e^	18.55 ^e–g^	4.14 ^g–i^	30.55 ^bc^	16.38 ^f–h^	82.77 ^a–c^	0.10 ^de^	8.78 ^bc^	34.24 ^bc^	5.47 ^f^
KIS_18	2.12 ^c–f^	4.35 ^fg^	2.32 ^d–h^	20.87 ^ef^	6.11 ^a^	21.57 ^ef^	13.70 ^h–j^	56.87 ^fg^	0.04 ^ij^	5.58 ^f–h^	21.22 ^h^	3.09 ^gh^
KIS_19	2.54 ^ab^	4.47 ^fg^	2.14 ^f–i^	20.68 ^ef^	3.19 ^j^	18.68 ^ef^	13.55 ^h–j^	58.31 ^fg^	0.11 ^d^	7.52 ^de^	27.93 ^d–f^	1.32 ^j–l^
Range	1.52–2.73	4.00–6.72	1.81–3.56	14.81–33.02	3.03–6.42	16.25–69.90	11.44–30.14	40.67–92.28	0.02–0.37	4.74–11.26	20.15–90.27	0.95–10.92
Mean ± SD	2.26 ± 0.29	4.90 ± 0.59	2.45 ± 0.37	22.31 ± 4.29	4.69 ± 0.91	27.99 ± 10.42	17.04 ± 4.62	64.59 ± 13.34	0.09 ± 0.07	7.12 ± 1.62	30.85 ± 13.73	4.25 ± 2.81
CV (%)	12.71	12.12	15.05	19.21	19.31	37.22	27.14	20.65	75.70	22.76	44.51	66.01

Data are means (*n* = 3). Mean values with different letters (a–l) in a column are significantly different (*p* < 0.05). DW—dry weight.

**Table 4:** Nutrient comparison of results of 19 analysed French beans (fresh, raw pods) expressed per 100 g fresh weight, with data from USDA and EuroFIR databases.

FCD Source	Country	ID/Sample	Water	Protein	Ash	Fat	OH	Energy	Mg	P	Ca	K	Na	Mn	Fe	Cu	Zn	Mo
			g	g	g	g	g	kcal	mg	mg	mg	mg	mg	mg	mg	mg	mg	μg
USDA	USA	2,346,400	89.7	1.97	0.68	0.28	7.41	40.0	28.2	41.0	40.0	290	2.5	0.33	0.65	0.10	0.35	41.6
EuroFIR	Italy	35,844	90.5	2.10	na	0.10	na	18.2	na	48.0	35.0	280	2.0	na	0.90	na	na	na
	Poland	41,023	89.1	2.40	0.70	0.20	7.60	26.0	22.0	44.0	65.0	264	6.0	0.22	1.10	0.10	0.61	na
	Slovenia	140,877	91.8	1.58	0.61	0.06	5.98	na	22.0	33.7	37.1	189	na	0.24	0.92	0.04	0.23	13.0
	Serbia	154,448	87.0	5.00	1.10	1.00	na	48.3	21.0	48.0	40.0	74	5.0	na	1.00	0.08	0.60	na
	Czech Republic	161,196	89.7	2.30	0.70	0.20	7.10	32.0	25.0	42.0	59.0	251	5.0	na	1.10	na	na	na
	Estonia	181,637	91.0	1.70	0.70	0.10	6.40	27.0	26.0	40.0	51.0	284	na	0.25	1.20	0.07	0.40	na
	Portugal	185,574	90.0	1.90	0.98	0.30	na	32.0	17.0	35.0	40.0	250	2.0	na	0.70	na	0.20	na
	United Kingdom	212,355	91.3	2.10	na	0.40	na	24.4	25.0	38.0	52.0	286	na	0.31	1.04	0.06	0.40	na
	Netherlands	216,072	91.4	2.30	0.70	0.20	na	25.1	30.0	50.0	70.0	250	3.0	na	0.80	0.06	0.25	na
	Norway	223,027	92.0	1.70	na	0.10	na	27.2	26.0	40.0	51.0	284	na	na	1.20	0.07	0.40	na
	Denmark	224,232	90.5	1.90	0.73	0.23	6.61	30.2	17.0	39.0	60.0	237	1.7	0.25	1.00	0.06	0.39	20.0
FCD summary		Mean	90.3	2.25	0.77	0.26	6.85	30.0	23.6	41.6	50.0	245	3.4	0.27	0.97	0.07	0.38	24.9
		SD	1.3	0.87	0.15	0.24	0.57	7.8	4.0	4.9	11.2	58	1.6	0.04	0.17	0.02	0.13	12.2
Study results		KIS-1	91.3	1.92	0.74	0.07	7.86	39.8	26.8	53.7	50.4	286	0.4	0.15	0.88	0.09	0.31	2.6
		KIS-2	91.7	2.43	0.78	0.01	7.80	41.0	24.9	58.1	41.7	309	0.3	0.27	0.70	0.10	0.33	8.2
		KIS-3	92.1	2.58	0.94	0.06	7.96	42.7	24.3	70.8	59.2	366	0.3	0.21	0.73	0.08	0.30	3.9
		KIS-4	92.4	2.72	1.07	0.12	8.16	44.6	25.7	75.5	73.4	413	0.4	0.20	0.79	0.07	0.29	2.8
		KIS-5	91.5	2.30	0.77	0.08	7.62	40.5	24.7	63.0	55.8	301	0.2	0.22	0.57	0.07	1.01	3.3
		KIS-6	86.1	1.64	0.37	0.05	4.74	26.0	18.8	33.6	37.0	120	0.1	0.10	0.50	0.08	0.21	9.6
		KIS-7	92.3	2.46	0.93	0.01	8.56	44.2	32.3	56.0	74.0	333	0.4	0.22	0.59	0.08	0.37	7.6
		KIS-8	88.4	1.49	0.48	0.08	6.03	30.8	21.7	38.4	44.2	176	0.2	0.17	0.47	0.06	0.25	7.0
		KIS-9	86.8	1.52	0.36	0.05	5.22	27.4	16.4	37.0	25.3	118	0.2	0.10	0.67	0.07	0.24	13.0
		KIS-10	72.6	0.68	0.19	0.03	2.54	13.2	7.7	15.3	13.7	76	0.1	0.04	0.16	0.02	0.09	8.7
		KIS-11	90.2	1.81	0.76	0.02	7.06	35.7	26.4	45.3	61.7	283	0.7	0.13	0.56	0.05	0.27	11.1
		KIS-12	93.0	2.92	0.76	0.16	9.29	50.3	34.1	76.7	74.9	249	0.4	0.41	1.04	0.08	0.38	2.9
		KIS-13	91.1	1.95	0.64	0.07	7.64	39.0	25.2	50.8	61.7	235	0.3	0.29	0.78	0.11	0.30	2.3
		KIS-14	88.6	1.64	0.45	0.07	6.11	31.6	14.1	44.3	33.7	175	0.3	0.11	0.63	0.06	0.20	2.2
		KIS-15	92.6	2.72	0.79	0.15	8.69	47.0	34.5	64.6	65.5	295	0.4	0.29	1.14	0.09	0.47	1.3
		KIS-16	92.5	3.05	0.74	0.11	8.47	47.1	34.6	85.7	56.8	275	0.4	0.25	1.02	0.09	0.50	1.6
		KIS-17	87.9	1.83	0.44	0.01	5.47	29.3	17.6	41.0	34.3	154	0.3	0.14	0.69	0.07	0.28	7.0
		KIS-18	88.8	1.46	0.51	0.08	6.29	31.7	18.9	38.7	54.5	186	0.2	0.12	0.51	0.05	0.19	3.7
		KIS-19	86.3	1.27	0.39	0.04	5.13	25.9	18.4	32.5	23.2	150	0.1	0.10	0.42	0.05	0.20	1.6
		Mean	89.3	2.02	0.64	0.07	6.88	36.2	23.5	51.6	49.5	237	0.3	0.18	0.68	0.07	0.33	5.3
		SD	4.5	0.62	0.23	0.04	1.67	9.2	7.1	17.6	17.6	90	0.1	0.09	0.23	0.02	0.19	3.5
Complementarity of study results vs. FCDs (%)		Min	83.4	42.9	30.7	20.0	42.5	72.7	45.3	45.5	39.1	102.2	6.6	20.0	24.0	66.3	44.2	9.7
		Max	101.1	61.0	97.0	15.9	122.2	104.2	115.4	171.4	107.0	142.4	11.4	123.4	95.2	111.8	166.4	31.3
		Mean	98.8	90.0	83.0	25.9	100.4	120.5	99.9	124.2	99.0	96.7	8.6	69.0	69.8	103.4	85.2	21.3

FCD, food composition database; OH, carbohydrates; na, not available; SD, standard deviation.

**Table 5:** List of French beans (*Phaseolus vulgaris* L.) grown in 2021 and included in this nutritional trait study.

Sample	Variety	Plant: Growth Type	Pod: Colour	Pod: Cross-Section Shape	Group *	Statistical Region/Growing Site	Altitude
KIS_1	Merveille de Piemonte	dwarf	yellow with violet flecks	narrow elliptic	I	Goriška/Ajdovščina	137 m
KIS_2	Parker	dwarf	green	circular	II	Goriška/Ajdovščina	131 m
KIS_3	Telemaco	dwarf	yellow	narrow elliptic	III	Goriška/Ajdovščina	131 m
KIS_4	Minosse	dwarf	green	narrow elliptic	IV	Goriška/Ajdovščina	131 m
KIS_5	Marconi Nano	dwarf	green	narrow elliptic	IV	Goriška/Šmarje-Ajdovščina	215 m
KIS_6	Topcrop	dwarf	green	elliptic–ovate	V	Osrednjeslovenska/Vrhnika	295 m
KIS_7	Berggold	dwarf	yellow	elliptic–ovate	VI	Osrednjeslovenska/Horjul	355 m
KIS_8	Capitano	dwarf	yellow	narrow elliptic	III	Osrednjeslovenska/Vrhnika	315 m
KIS_9	Altea	dwarf	green	narrow elliptic	IV	Osrednjeslovenska/Ljubljana	303 m
KIS_10	local population	dwarf	yellow	circular	VII	Koroška/Ribnica na Pohorju	715 m
KIS_11	Helios	dwarf	yellow	elliptic–ovate	VI	Posavska/Senovo	230 m
KIS_12	Telemaco	dwarf	green	narrow elliptic	IV	Gorenjska/Podnart	404 m
KIS_13	Bina	dwarf	green	narrow elliptic	IV	Obalno-kraška/Strunjan	10 m
KIS_14	Telemaco	dwarf	yellow	narrow elliptic	III	Obalno-kraška/Strunjan	10 m
KIS_15	local population	climbing	violet	elliptic–ovate	VIII	Goriška/Livek-Kobarid	828 m
KIS_16	local population	climbing	green with violet flecks	narrow elliptic	IX	Goriška/Livek-Kobarid	828 m
KIS_17	local population	dwarf	yellow	elliptic–ovate	VI	Savinjska/Šmartno ob Paki	313 m
KIS_18	local population	dwarf	violet	circular	X	Savinjska/Šmartno ob Paki	313 m
KIS_19	KIS Amand	dwarf	yellow	narrow elliptic	III	Osrednjeslovenska/Jablje-Mengeš	302 m

* see also Figure 6.

## Data Availability

Data are contained within the article.
